# Enhanced visible-light-driven photocatalytic performance for degradation of organic contaminants using PbWO_4_ nanostructure fabricated by a new, simple and green sonochemical approach

**DOI:** 10.1016/j.ultsonch.2020.105420

**Published:** 2020-12-29

**Authors:** Sahar Zinatloo-Ajabshir, Mahin Baladi, Masoud Salavati-Niasari

**Affiliations:** a Department of Chemical Engineering, University of Bonab, P.O. Box. 5551761167, Bonab, Iran; b Institute of Nano Science and Nano Technology, University of Kashan, Kashan, P. O. Box. 87317-51167, IR, Iran

**Keywords:** Lead tungstate, Nanocatalyst, Green chemistry, Photodegradation, Ultrasound irradiation

## Abstract

Water contamination has turned into a critical global concern that menaces the entire biosphere and has a notable effect on the lives of living beings and humans. As a proper and environmentally friendly solution, visible-light photocatalysis technology has been offered for water contamination removal. There is a strong interest in the design of the efficient catalytic materials that are photoactive with the aid of visible light. Herein, to fabricate a highly efficient photocatalyst for removal of organic pollution in water, a facile and swift sonochemical route employed for creation of the spindle shaped PbWO_4_ nanostructure with the aid of an environmentally friendly capping agent (maltose) for the first time. To optimize the efficiency, dimension and structure of lead tungstate, various effective factors such as time, dose of precursors, power of ultrasound waves and kind of capping agents were altered. The attributes of PbWO_4_ samples were examined with the aid of diverse identification techniques. The produced lead tungstate samples in role of visible-light photocatalyst were applied to remove organic pollution in water. The kinds of pollutants, dose and type of catalyst were examined as notable factors in the capability to eliminate contaminants. Very favorable catalytic yield and durability were demonstrated by spindle-shaped PbWO_4_ nanostructure (produced at power of 60 W for 10 min and with usage of maltose). Usage of ultrasonic irradiation could bring to improvement of catalytic yield of PbWO_4_ to 93%. Overall, the outcomes could introduce the spindle-shaped PbWO_4_ nanostructure as an efficient substance for eliminating water contamination under visible light.

## Highlights

• Spindle shaped PbWO_4_ nanostructure with enhanced photocatalytic activity was obtained by facile and green sonochemical way for the first time.
• Maltose as a new capping agent was utilized.
• Sonication time, dose of precursors, power of ultrasound waves and kind of capping agent were optimized.
• The made lead tungstate samples in role of visible-light photocatalyst were applied to remove organic pollution in water.
• Usage of ultrasonic irradiation has remarkable effect on performance of PbWO_4_ photocatalyst for decomposition.

## 1 Introduction

As a proper and environmentally friendly solution to address a critical global concern that could affect the entire globe, visible-light photocatalysis technology has been proposed to eliminate water contamination [1]. In this approach, the creation of reactive oxygen species to remove water contaminants can happen with the aid of photocatalyst possessing desirable band gap through advanced oxidation processes [2], [3], [4], [5]. One of the most notable areas of research pursued by scientists is the design of efficacious catalytic materials that are activated with the aid of visible light [6], [7], [8]. The reason for this is the maximal usage of free and plentiful solar energy for water contamination removal.

Lead tungstate has aroused very notable interest due to its wide range of uses in scintillators, amplifiers, photocatalysis and lasers [9], [10]. Lead tungstate structures have been prepared applying various approaches such as co-precipitation, hydrothermal, solid-state reaction and Bridgman route [11], [12], [13], [14]. Today, the sonochemical pathway is applied as a helpful synthesis procedure to create a range of nanoscale compounds with various shapes [15], [16], [17]. Ultrasound is employed as a swift procedure for various tasks such as architectural control of nanostructures and dispersion of materials [18]. Cavitation created with the aid of ultrasound waves may bring to favorable and specific structures with high uniformity at the nanoscale. Owing to hot-spot theory, creation of excessively high temperatures and release of immense energies can be happened within sonochemical cavitation that can be favorable for a wide range of chemical reactions.

Nowadays, the architectural control of nanoscale compounds with very favorable shape and dimension is a momentous aim for researches because their features and yield are dependent on shape and dimension [19], [20], [21], [22]. Green chemistry-based pathway defining as the design and usage of procedures to create a range of nanoscale compounds with the help of bioproducts, has aroused very notable interest [23], [24]. The reason for this is the wide availability, non-toxicity and renewability of substances that are utilized to create nanoscale compounds with green chemistry-based pathway, being safe for environment and humans [25], [26]. This paper offers a new pathway through ultrasound-aided fabrication of spindle shaped PbWO_4_ nanostructure with the help of an environmentally friendly capping agent (maltose) for the first time. To the best of our knowledge, usage of maltose has never been reported for the creation of PbWO_4_ nanostructure under ultrasound waves. We decided to apply maltose due to its non-toxicity and wide availability. We explored the role of various effective factors such as time, dose of precursors, power of ultrasound waves and kind of capping agents on the uniformity, efficiency, dimension and structure of lead tungstate. The attributes of PbWO_4_ samples were examined with the aid of diverse identification techniques. Further, we applied the prepared lead tungstate samples in role of visible-light photocatalyst to remove organic pollution in water. The yield of lead tungstate in role of visible-light photocatalyst rarely was examined. We examined the role of kind of pollutants, dose and type of catalyst as notable factors in contaminant removal capability.

## 2 Experimental

### 2.1 Sonochemical synthesis of spindle shaped PbWO_4_ nanostructure

All substances such as fructose (C_6_H_12_O_6_), sodium tungstate dihydrate (Na_2_WO_4_·2H_2_O), maltose (C_12_H_22_O_11_), lead(II) acetate trihydrate (Pb(CH_3_COO)_2_·3H_2_O), starch (C_6_H_10_O₅)n and glucose (C_6_H_12_O_6_) were utilized to create PbWO_4_ samples, were bought from Merck. The ultrasound-aided pathway to prepare PbWO_4_ samples was applied. Ultrasonic irradiation was performed utilizing a multiwave ultrasonic generator equipped with a converter/transducer and titanium oscillator (horn), 12.5 mm in diameter (MPI Ultrasonics; welding, 1000 W, 20 kHz, Switzerland). At initial, 25 ml of aqueous solution comprising Na_2_WO_4_·2H_2_O was added to 25 ml of aqueous solution comprising Pb(CH_3_COO)_2_·3H_2_O and environmentally friendly capping agent (maltose) under ultrasound waves with power of 60 W for 10 min (molar ratio of Pb:W:maltose = 1:1:1). Afterward, the created white sample was washed (with ethanol and distilled H_2_O) and air-dried (sample 11). We explored the role of various effective factors such as time, dose of precursors, power of ultrasound waves and kind of capping agents on the uniformity, efficiency, dimension and structure of lead tungstate. Experimental details are observed in Table 1.

**Table 1:** The preparation conditions for various lead tungstate samples.

Sample no	Power (W)	Ultrasonic time (min)	Dose of lead precursor (mol)	Green capping agent	Figure of FESEM images
1	60	10	0.01	–	1a and b
2	30	10	0.01	–	1c and d
3	90	10	0.01	–	1e and f
4	60	5	0.01	–	2a and b
5	60	20	0.01	–	2c and d
6	60	10	0.04	–	3a and b
7	60	10	0.08	–	3c and d
8	60	10	0.16	–	3e and f
9	60	10	0.01	Fructose	4a and b
10	60	10	0.01	Glucose	4c and d
11	60	10	0.01	Maltose	4e and f
12	60	10	0.01	Amylum	4 g and h
13	–	–	0.01	–	6a and b

### 2.2 Characterization

TEM analysis was utilized to check the shape and dimension of the created PbWO_4_ sample on a Philips CM30 TEM. Optical features of the spindle shaped PbWO_4_ nanostructure were checked applying spectrophotometer (Shimadzu, UV-2550, Japan). The shape, structure and elemental composition of the created PbWO_4_ samples were explored utilizing MIRA3-TESCAN FESEM. The phase purity of the spindle shaped PbWO_4_ nanostructure was explored with a diffractometer of Philips Company. A vibrating sample magnetometer (VSM, Meghnatis Kavir Kashan Co., Kashan, Iran) was applied to explore the magnetism of the spindle shaped PbWO_4_ nanostructure. FT-IR spectrometer (Magna-IR) was applied to explore the surface of the spindle shaped PbWO_4_ nanostructure.

### 2.3 Photocatalytic activity

The prepared lead tungstate samples in role of visible-light photocatalyst were applied to remove organic pollution in water. The contaminant (1 mg of erythrosine or Acid Black 1 or methyl violet) solution with defined quantity of lead tungstate sample in role of visible-light photocatalyst were mixed and aerated within 1/2h in darkness for the equilibrium of the adsorption of contaminant upon the surface of lead tungstate sample. Subsequent, each mixture was irradiated with 125 W Osram lamp [8]. From following relation was employed to specify the contaminant (erythrosine or Acid Black 1 or methyl violet) decomposition rate:

(1) $$D.P.\left(t\right)=\frac{A_{0}-A_{t}}{A_{0}}\times 100$$

A_0_ and A_t_ mention the initial and ultimate absorbance for contaminant (erythrosine or Acid Black 1 or methyl violet) [8].

## 3 Results and discussion

Here, we offer a new pathway through ultrasound-aided fabrication of PbWO_4_ nanostructure with the help of an environmentally friendly capping agent. We explored the role of different effective factors such as time, dose of precursors, power of ultrasound waves and kind of capping agents on the uniformity, efficiency, dimension and structure of lead tungstate.

### 3.1 Morphology investigation

In sonochemistry, the power and time of ultrasound are examined as two efficient instrumental variables that can affect the features of products in terms of size and morphology [27]. Additionally, experimental variables such as the precursor dose and the kind of capping agent can affect the features of the products. Thus, optimization of these two kinds of variables seems necessary to achieve a product with very desirable features.

**Fig. 1:**
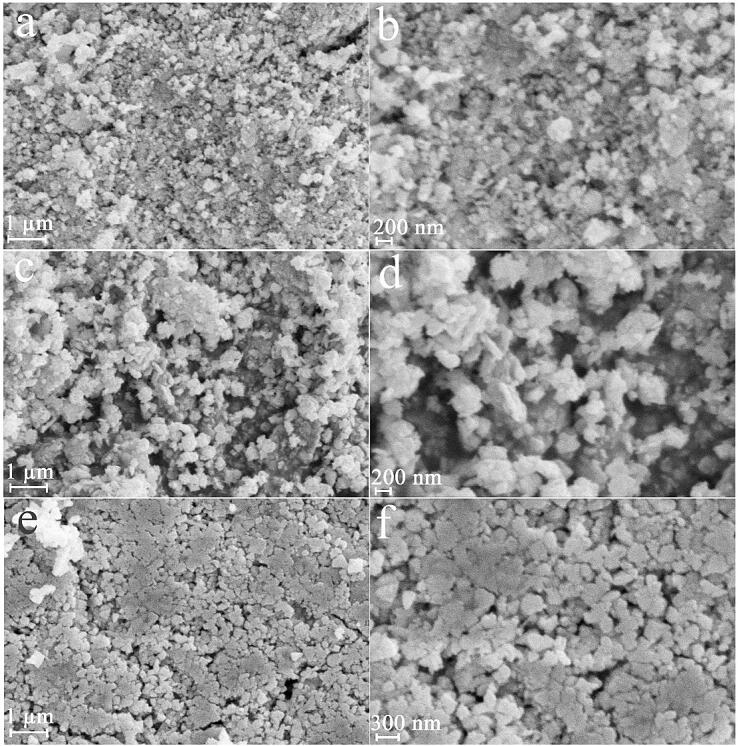
FESEM images of samples 1, 2 and 3 produced with 60 (a and b), 30 (c and d) and 90 W (e and f).

To explore the role of power of ultrasound waves on lead tungstate, samples 1, 2 and 3 were fabricated with 60, 30 and 90 W, correspondingly (see Fig. 1a-f). We observed that the alteration in power of ultrasound waves from 60 W to 30 and 90 W can bring to the change in features of lead tungstate in terms of size and uniformity. Very proper power to create the regular and suitable lead tungstate nanostructure can be 60 W (Fig. 1a and b). It is found in Fig. 1c-f, the irregular agglomerated structures could be created with usage of other powers (90 and 30 W). It seems that enhancing the power of ultrasound from 30 to 60 W can be effective in further collapse of the cavitation bubble, resulting in a stronger shock wave, which can hinder the agglomeration [28]. On the other hand, increasing the power of ultrasound can accelerate thermodynamic stability via the growth of primary nuclei, and thus particles can agglomerate owing to the release of more energy in less time [27]. Hence, very desirable ultrasonic power to achieve lead tungstate nanostructure with a high uniformity, 60 W was selected.

**Fig. 2:**
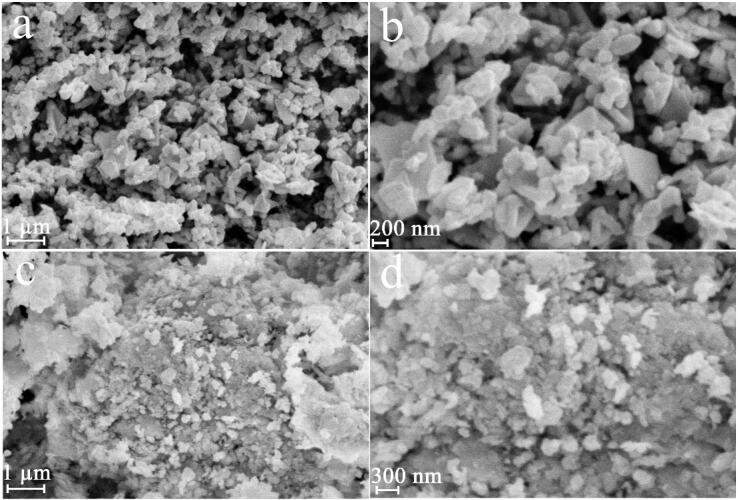
FESEM images of samples 4 and 5 produced with 60 W within 5 (a and b) and 20 (c and d) min.

Further, to explore the role of ultrasonic time on lead tungstate features, samples 4 and 5 were fabricated with 60 W for 5 and 20 min (see Fig. 2a-d). The shapeless structures can be created within 5 min. By enhancing the ultrasound time to 10 min, the regular and suitable lead tungstate nanostructure was created, which could indicate the positive effect of enhancing the ultrasound time (Fig. 1a and b). Thus, it was assumed that enhancing this variable for up to 20 min could reduce the particle size and improve the uniformity, but the outcomes demonstrated that irregular agglomerated micro/nanostructures were created during this time (Fig. 2c and d). By changing the ultrasound time from 5 to 10 min, energy is continuously added to the reaction system and can inhibit the growth of lead tungstate nanostructures [29]. It has been shown that by applying sonication, the primary nanoparticles can dissolve and grow into larger crystals. Crystal dissolution and growth are two parallel processes that occur [30]. Thus, by changing the ultrasound time from 5 to 10 min, the growth of the lead tungstate nanostructure may not be optimal in terms of energy. Instead, dissolving the lead tungstate nanostructure created over 5 min may be energetically desirable [31]. Ostwald ripening process may be reason for creation of the irregular agglomerated micro/nanostructures with the alteration in time from10 to 20 min. It seems that lead tungstate nanostructures created owing to high surface energy (induced with reduction in size to nanometer scales) [27], [32] can act as primary nuclei and cause the growth process to occur via Ostwald ripening process, resulting in the irregular agglomerated micro/nanostructures produced by applying ultrasound in more time [27]. Evidently, very convenient time to create the regular and suitable lead tungstate nanoparticles with tiny size can be 10 min (Fig. 1a and b).

**Fig. 3:**
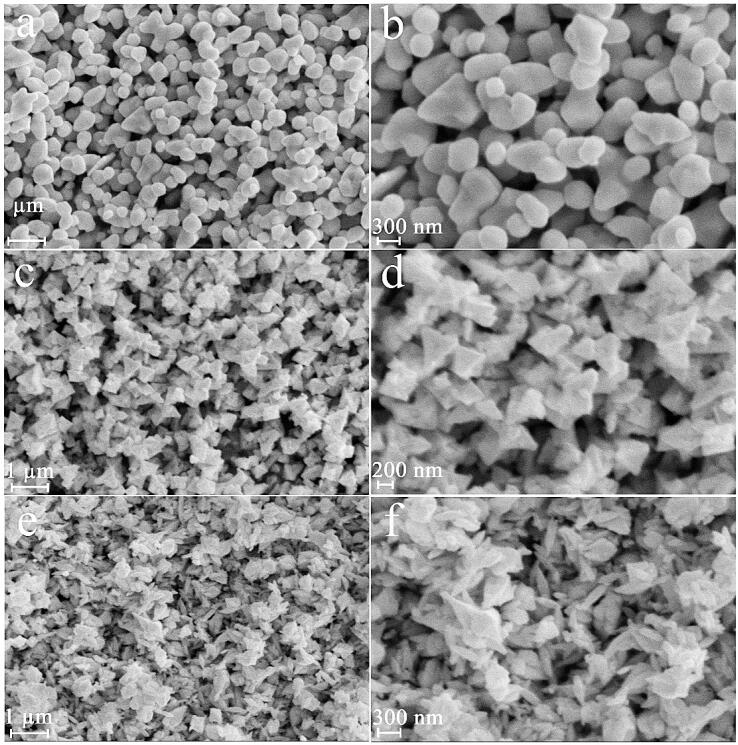
FESEM images of samples 6, 7 and 8 produced with 0.04 (a and b), 0.08 (c and d), 0.16 (e and f) mol of lead precursor (molar ratio of Pb:W = 1:1).

Also, we explored the role of dose of precursors on the uniformity and structure of lead tungstate and applied 0.04, 0.08, 0.16 mol of lead precursor (molar ratio of Pb:W = 1:1) for creation of samples 6, 7 and 8, correspondingly, with 60 W for 10 min. Less regular nanostructures, relatively homogeneous polygon nanostructures and scale-like nanostructures can be created with usage of 0.04 (Fig. 3a and b), 0.08 (Fig. 3c and d), 0.16 mol (Fig. 3e and f) of lead precursor. Obviously, very proper dose of lead precursor to fabricate the regular and suitable lead tungstate nanoparticles with tiny size can be 0.01 mol (Fig. 1a and b).

**Fig. 4:**
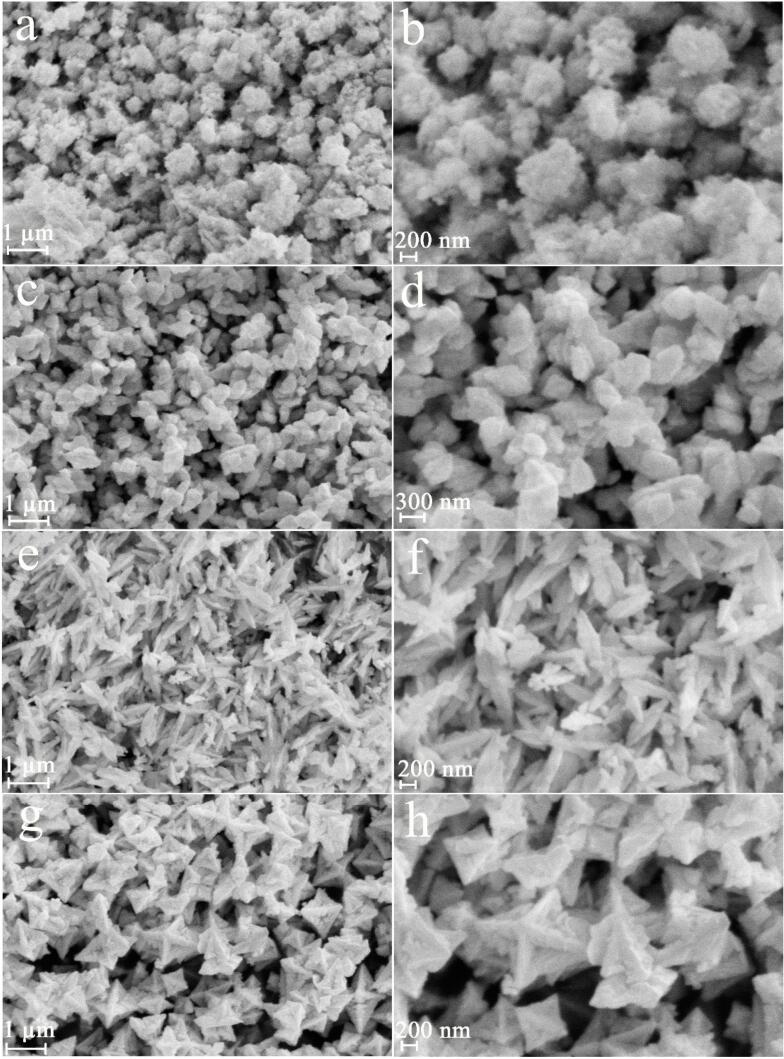
FESEM images of samples produced with fructose (a and b), glucose (c and d), maltose (e and f) and amylum (g and h).

To explore the role of kind of capping agent on lead tungstate features, samples 9, 10, 11 and 12 were fabricated with fructose, glucose, maltose and amylum (see Fig. 4a-h). Cluster-like nanostructures (Fig. 4a and b), polygon-like nanostructures (Fig. 4c and d), very homogeneous spindle shaped PbWO_4_ nanostructures (Fig. 4e and f) and star-like micro/nanostructures (Fig. 4g and h) can be created with usage of fructose, glucose, maltose and amylum. It seems that the environmentally friendly capping agents (fructose, glucose, maltose and amylum) are able to the inducement of initial nanoparticles for assembly in the specified directions, being advantageous for creation of various structures. Evidently, usage of maltose as environmentally friendly capping agent brings to creation of spindle shaped PbWO_4_ nanostructure with great uniformity under ultrasound irradiation. Further, we can conclude that architectural control of lead tungstate can be made possible with usage of diverse capping agents under ultrasound irradiation.

**Fig. 5:**
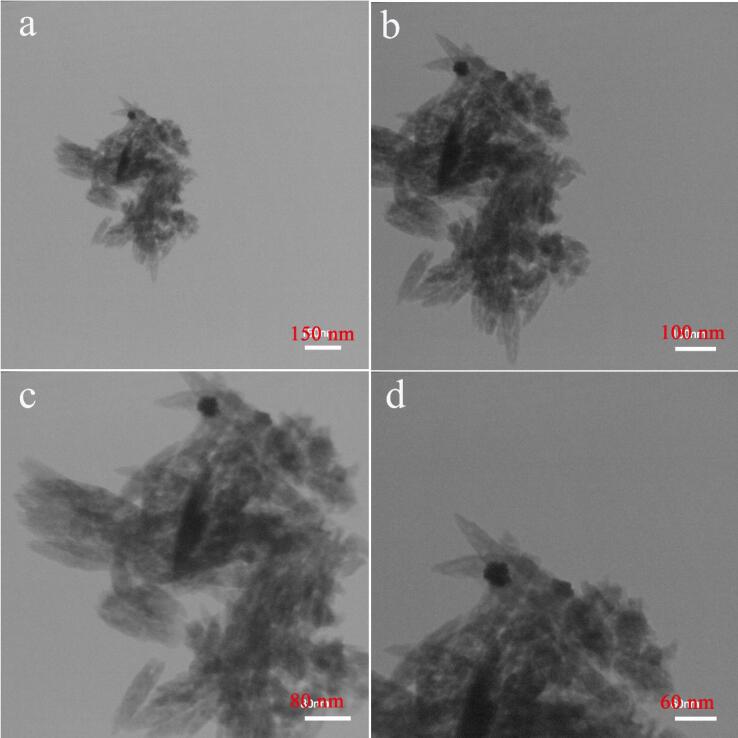
TEM images of PbWO_4_ nanostructures produced at power of 60 W for 10 min and with usage of maltose (sample 11).

It is found in Fig. 5a-d, the successful fabrication of spindle shaped PbWO_4_ nanostructures has been corroborated with the aid of TEM data. Sample 11 displays spindle-like morphology. We considered it as the optimal structure having very proper uniformity.

### 3.2 Formation mechanism of spindle shaped PbWO_4_ nanostructure

**Fig. 6:**
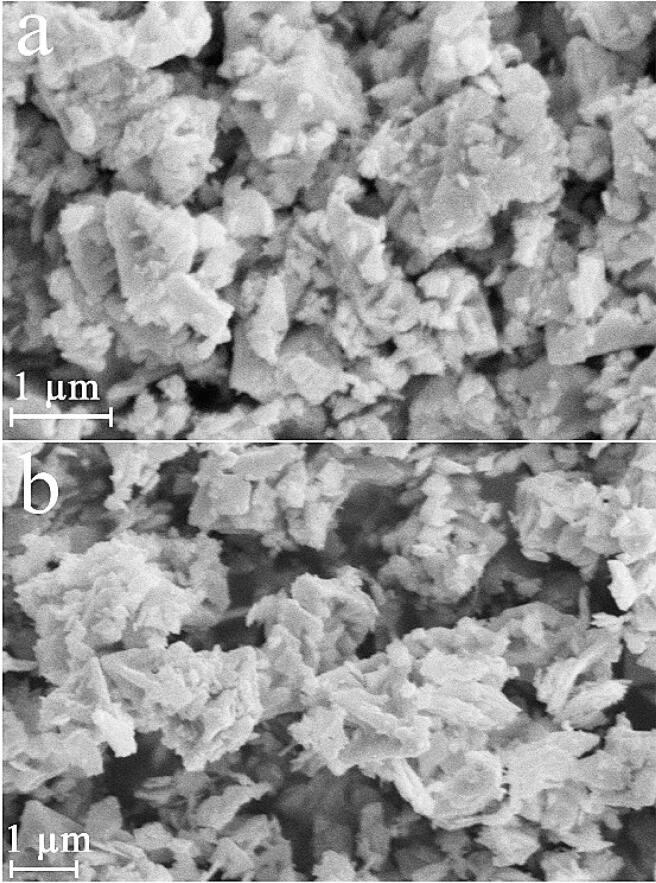
FESEM images of sample 13 produced through vigorous stirring within 40 min in presence of maltose.

**Scheme 1:**
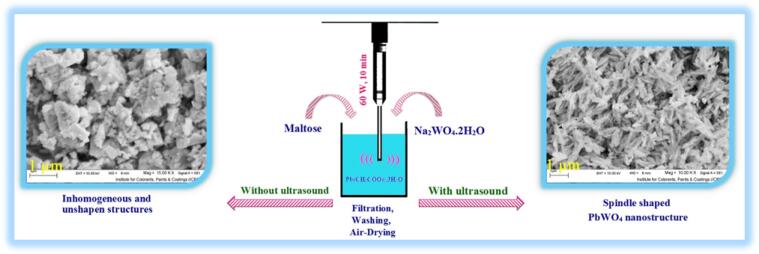
Schematic diagram of creation of spindle shaped PbWO_4_ nanostructure and impact of application of ultrasound waves on the size and shape of PbWO_4._

To explore the role of ultrasonic irradiation, sample 13 was produced through vigorous stirring within 40 min in presence of maltose. Sample 13 displays the inhomogeneous and unshapen structures (see Fig. 6a and b). Ultrasound is employed as a swift tool for various tasks like architectural control of nanostructures [18]. Cavitation created with the aid of ultrasound waves may bring to favorable and specific nanoscale structures with high uniformity. Owing to hot-spot theory, creation of excessively high temperatures and release of immense energies can be happened within bubbles collapse that can be favorable to conversion of massive structures to tiny particles [33]. Thus, we can conclude that ultrasound irradiation can be very advantageous in architectural control of lead tungstate (see Scheme 1).

As noted above, spindle shaped PbWO_4_ nanostructure with great uniformity was sonochemically fabricated with usage of maltose. The simultaneous impact of ultrasonic ultrasound waves and maltose as an environmentally friendly capping agent may be reason to create very homogeneous spindle shaped PbWO_4_ nanostructure. Thus, special propulsion conditions resulting from sonochemical cavitation can be favorable to simultaneous gelatinization of maltose and creation of spindle shaped PbWO_4_ nanostructure. The possible mechanism to create the spindle shaped PbWO_4_ nanostructure may be as:

Pb(CH_3_COO)_2_·3H_2_O _+_ H_2_O → 2CH_3_COO2^−^ + Pb^2+^

H_2_O + ultrasound waves → H^.^ + OH^.^

Na_2_WO_4_·2H_2_O + H_2_O → 2Na^+^ + WO_4_^2-^

Pb^2+^ + WO_4_^2-^ → PbWO_4_

H^.^ + OH^.^ → H_2_O

The sonochemical formation of the spindle shaped PbWO_4_ nanostructure may be taken place in two stages: the ultrasound-induced creation of the initial nuclei and afterwards ultrasound-induced assembly of the formed nuclei to make the spindle shaped PbWO_4_ nanostructure.

### 3.3 Structure and purity of spindle shaped PbWO_4_ nanostructure

**Fig. 7:**
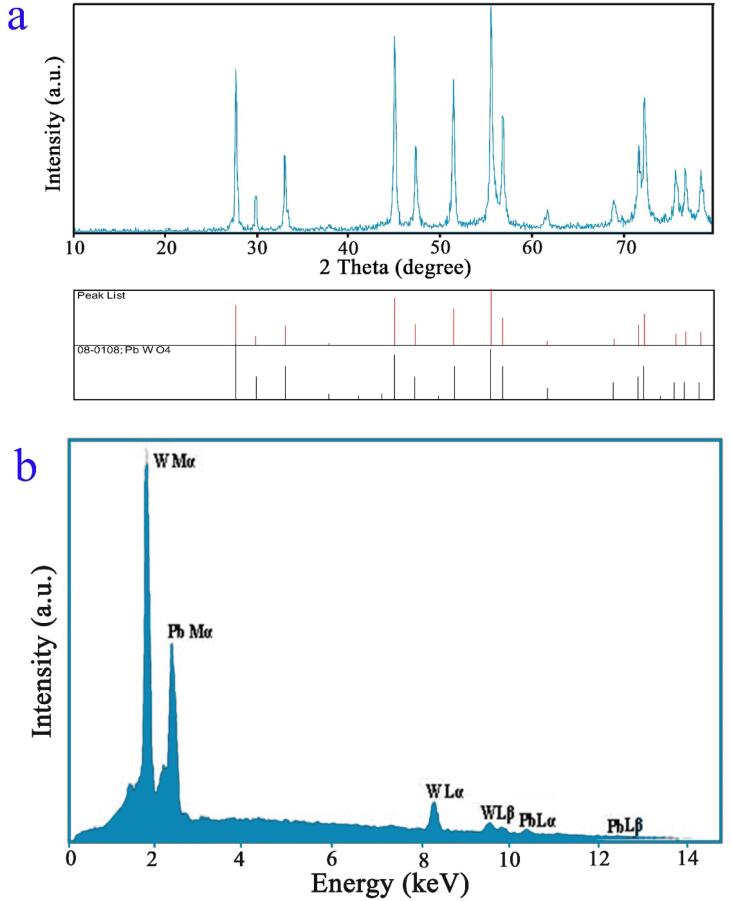
(a) XRD pattern and (b) EDS pattern of PbWO_4_ nanostructures produced at power of 60 W for 10 min and with usage of maltose (sample 11).

The successful formation of pure PbWO_4_ nanostructure (sample 11) was corroborated with XRD outcome (see Fig. 7a). The outcome is in full compliance with tetragonal phase lead tungsten oxide (JCPDS No. 08-0108). The crystallite size for the spindle-shaped PbWO_4_ nanostructure was estimated applying the Scherrer equation [5] to be near 38 nm. We found no characteristic diffraction band for impurity in XRD outcome, signifying purity of the spindle-shaped nanostructure.

Fig. 7b displays EDS outcome for checking purity of the spindle-shaped PbWO_4_ nanostructure. The elements are seen in the composition, denote creation of PbWO_4_. Sample 11 comprises lead and tungsten and this outcome corroborates XRD findings.

**Fig. 8:**
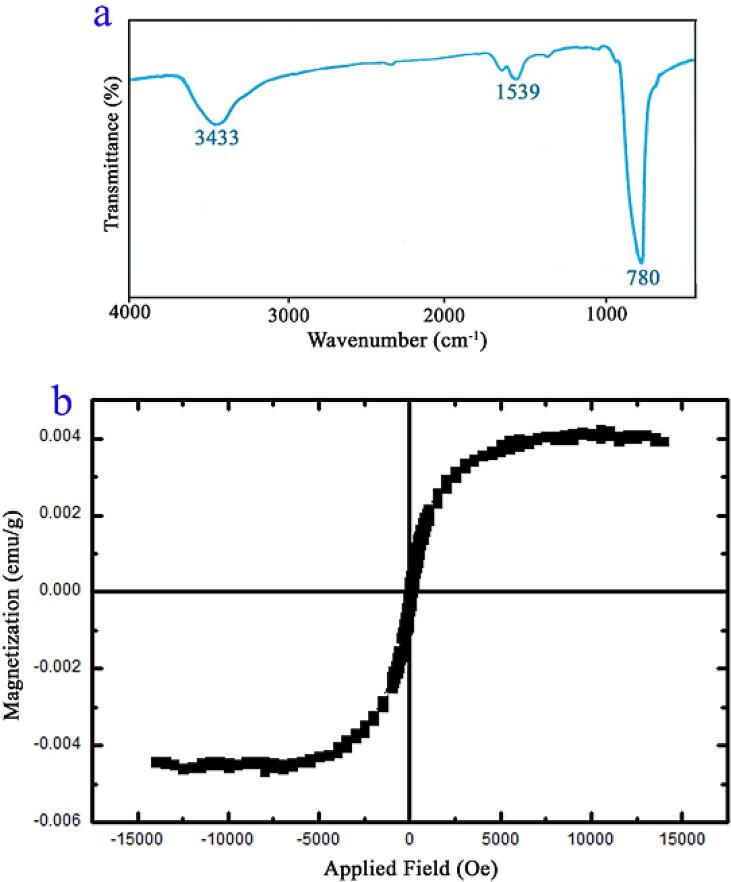
(a) FT-IR spectrm and (b) VSM outcome of PbWO_4_ nanostructures produced at power of 60 W for 10 min and with usage of maltose (sample 11).

The successful formation of pure PbWO_4_ nanostructure (sample 11) was also corroborated with FT-IR outcome (see Fig. 8a). The band near 780 cm^−1^ may be assigned to lead tungsten oxide [34]. The bands around 3433 and 1539 cm^−1^ may be ascribed to the physisorbed water [3].

### 3.4 Magnetism and optical property of spindle-shaped PbWO_4_ nanostructure

Fig. 8b gives VSM outcome for checking the magnetism of the spindle-shaped PbWO_4_ nanostructure. Obviously, sample 11 possesses proper magnetism (saturation magnetization = 0.0042 emu g^−1^) for simple recovery that is momentous in practical utilizations.

**Fig. 9:**
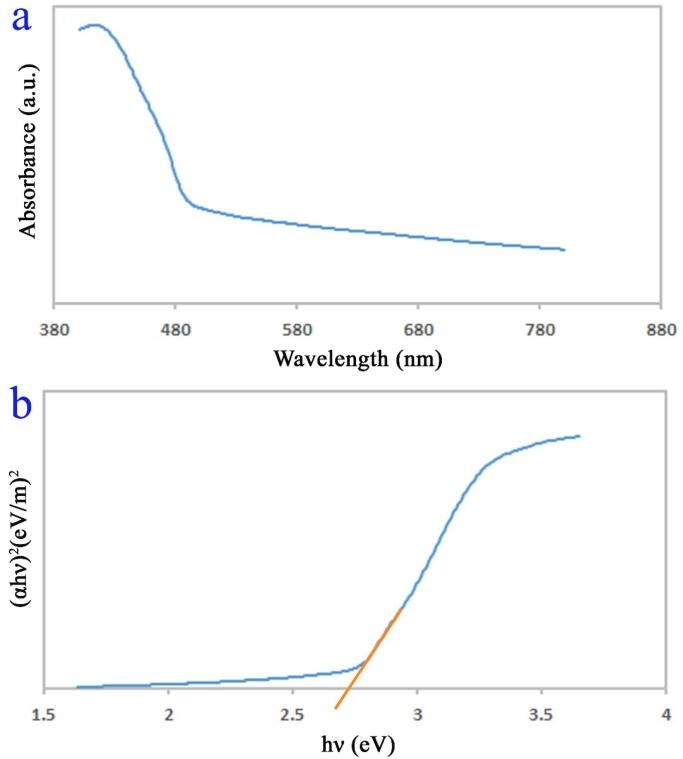
DRS spectrm (a), plot to determine the band gap (b) of PbWO_4_ nanostructures produced at power of 60 W for 10 min and with usage of maltose (sample 11).

The optical features of the spindl-shaped PbWO_4_ nanostructure (sample 11) were examined with DRS (see Fig. 9). An absorption band nearly 417 nm is seen. It is accepted that the energy gap is the notable parameter impacting photocatalytic yield. The determined energy gap for the spindle-shaped PbWO_4_ nanostructure from Tauc's plot [8] is of about 2.7 eV. Accordingly, this outcome corroborates easy activation of the spindle-shaped PbWO_4_ in the role of the nano-sized photocatalyst (by visible illumination) to remove water contamination.

### 3.5 Photocatalytic activity

**Fig. 10:**
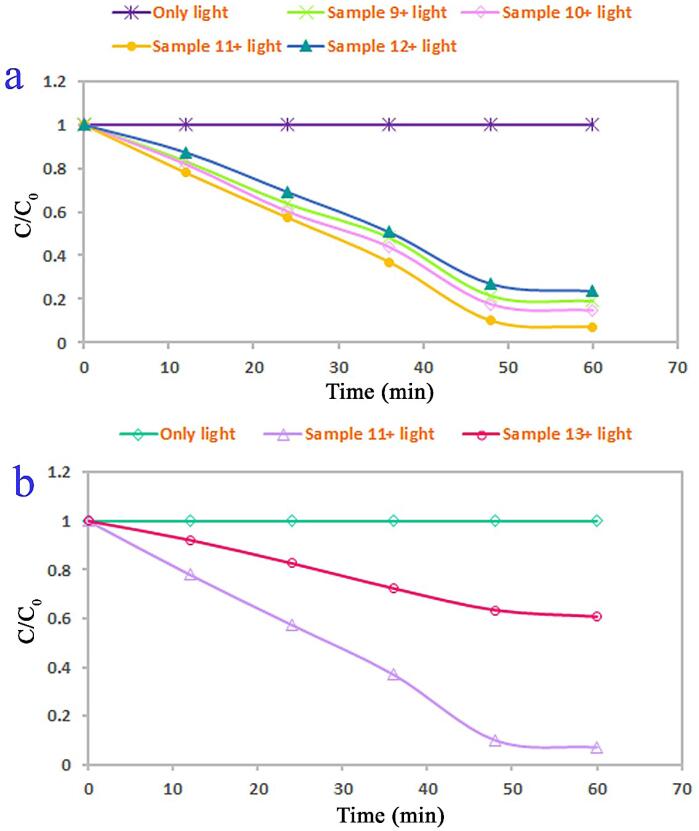
Photocatalytic decomposition of Acid Black 1 under visible light over PbWO_4_ structures (samples 9–13).

**Fig. 11:**
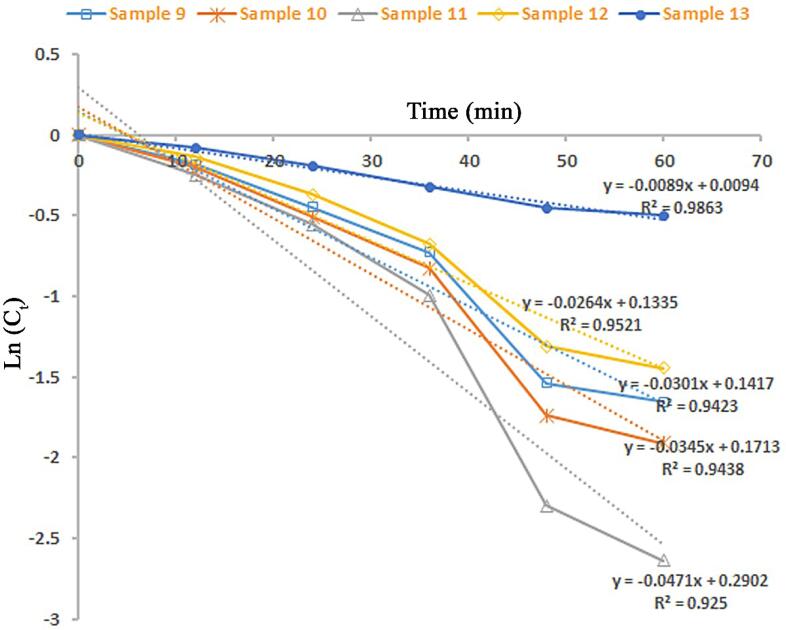
Reaction kinetics of photocatalytic decomposition of Acid Black 1 under visible light over PbWO_4_ structures (samples 9–13).

The produced lead tungstate samples in role of visible-light photocatalyst were applied to remove organic pollution such as Acid Black 1 in water. The role of utilization of ultrasound waves as well as kind of capping agent on lead tungstate efficiency (samples 9–13) was explored (see Fig. 10, Fig. 11). Insignificant catalytic decomposition of Acid Black 1 was occurred without usage of any lead tungstate samples. We found that, after 60 min of illumination, the decomposition amount of Acid Black 1 for the spindle-shaped PbWO_4_ nanostructure (sample 11) is about 93% and it denoted the most proper photocatalytic yield. In contrast, about 81, 86, and 77% of Acid Black 1 could be destructed with the help of the cluster-like nanostructures (sample 9), polygon-like nanostructures (sample 10), and star-like micro/nanostructures (sample 12). The yield of the spindle-shaped PbWO_4_ nanostructure (sample 11) is the more proper than the yield of the inhomogeneous and unshapen structures (sample 13). Sample 13 could eliminate about 40% of Acid Black 1 under the same condition. Additionally, the reaction kinetics of the produced lead tungstate samples were checked utilizing the pseudo-first-order mode [35]. It is found in Fig. 11, the rate constants (K) of 0.0301, 0.0345, 0.0471, 0.0264 and 0.0089 min^−1^ are for samples 9, 10, 11, 12 and 13. Evidently, the lead tungstate samples fabricated with utilization of ultrasound waves denotes a much proper photocatalytic yield than the sample 13 produced without utilization of ultrasound waves. Usage of ultrasonic irradiation could bring to improvement of catalytic yield of PbWO_4_ to 93%. From FESEM outcomes (see Fig. 4, Fig. 6), we can conclude that utilization of ultrasound waves and maltose as an environmentally friendly capping agent has very notable and efficacious impact on creation of very homogeneous spindle-shaped PbWO_4_ nanostructure with specific architecture as the most efficient photocatalyst for water contamination removal.

**Fig. 12:**
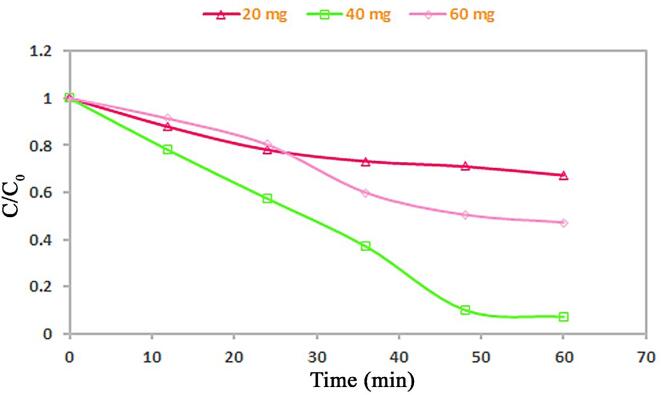
Influence of PbWO_4_ nanostructure dose on photocatalytic yield for elimination of Acid Black 1.

After type of catalyst, we explored the role of its dose as notable factor in contaminant removal capability (see Fig. 12). We found that alteration in catalyst dose from 20 to 40 mg, could bring to increment in decomposition yield (33 to 93%), but by utilization of 60 mg catalyst, 53% of Acid Black 1 could be destructed. The enhancement of surface area and thus improvement of the absorption of Acid Black 1 on the spindle-shaped PbWO_4_ nanostructure surface may be reason for increment in decomposition yield. In contrast, the nanostructure thickness as well as saturation of PbWO_4_ nanostructure layers may be reason for decrement of catalytic yield by utilization 60 mg catalyst. Consequently, very proper dose of the spindle-shaped PbWO_4_ nanostructure in role of catalyst can be 40 mg.

**Fig. 13:**
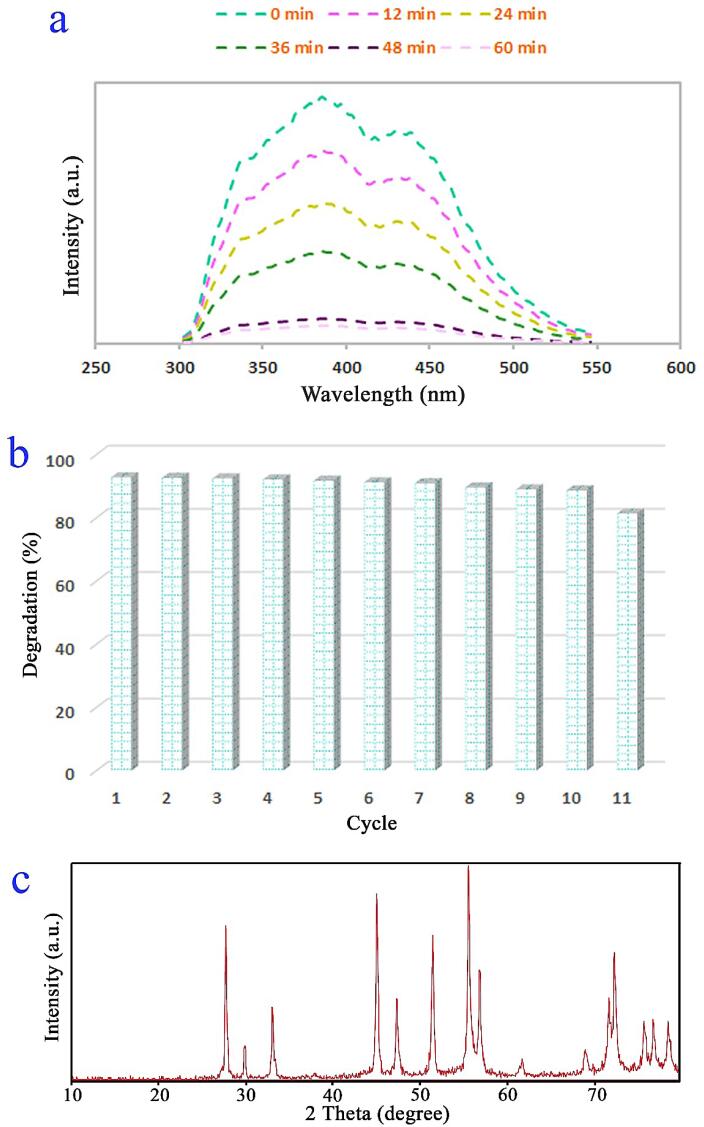
(a) Fluorescence spectral time scan of Acid Black 1 solution under visible light with the aid of PbWO_4_ nanostructure (sample 11), (b) 11 cycles of the decomposition of Acid Black 1 under the illumination of visible light over sample 11 and (c) XRD pattern of sample 11 after visible-light photocatalytic activity.

The spectral changes within the decomposition of Acid Black 1 with utilization of the spindle-shaped PbWO_4_ nanostructure as visible-light photocatalyt are given in Fig. 13a. Evidently, the elimination of Acid Black 1 is continuous. It is accepted that as a momentous attribute of nano-sized catalyst can be stability of it over several catalytic runs. Upon repeating the visible-light photocatalysis experiments, 82% of visible-light photocatalysis yield was achieved after eleven times of the spindle-shaped PbWO_4_ nanostructure reuse (see Fig. 13b). This outcome corroborates very proper durability of PbWO_4_ nanostructure. Fig. 13c gives XRD outcome for checking the consistency of the spindle-shaped PbWO_4_ nanostructure after catalytic experiment to eliminate Acid Black 1. It is obvious that XRD outcomes of the spindle shaped PbWO_4_ nanostructure before and after visible-light photocatalysis experiments are identical (see Fig. 7a and 13c).

The possible mechanism to eliminate Acid Black 1 with the help of the spindle-shaped PbWO_4_ nanostructure may be expressed as [17]:

Spindle shaped PbWO_4_ nanostructure + hν → Spindle shaped PbWO_4_ nanostructure* + e^−^ + h^+^

h^+^ + H_2_O → OH^.^

e^−^ + O_2_ → O_2_^−.^

OH^.^ + O_2_^−.^ + Acid Black 1 pollutant → Degradation products

**Fig. 14:**
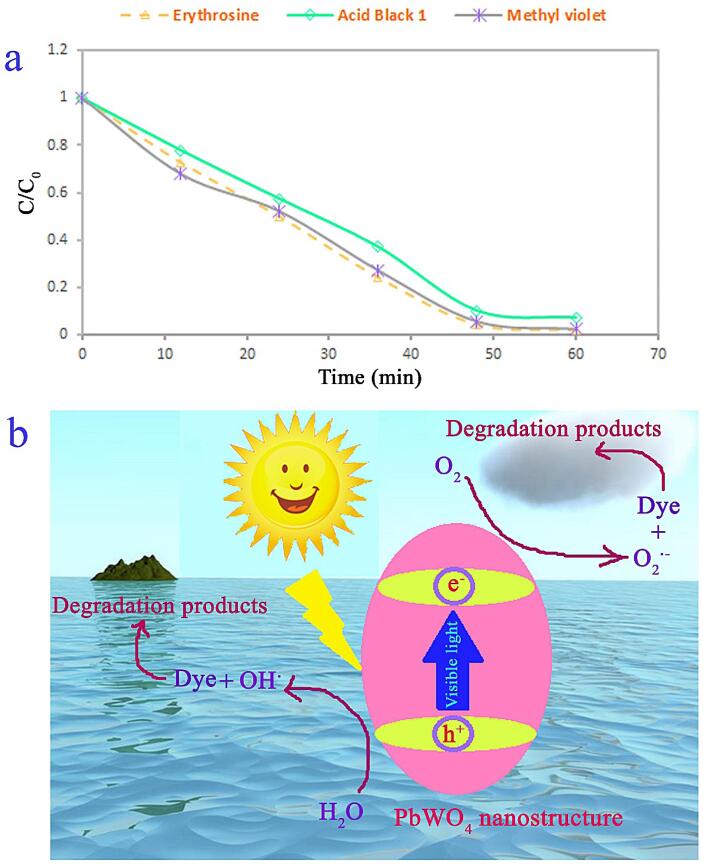
(a) Photocatalytic decomposition of organic pollution like erythrosine and methyl violet over PbWO_4_ nanostructure (sample 11) and (b) schematic diagram of photocatalytic process of PbWO_4_ nanostructure. (For interpretation of the references to colour in this figure legend, the reader is referred to the web version of this article.)

Visible-light photocatalytic ability of the spindle-shaped PbWO_4_ nanostructure was also explored to eliminate organic pollution like erythrosine and methyl violet in water (see Fig. 14). Yield of the spindle-shaped PbWO_4_ nanostructure to eliminate erythrosine and methyl violet is about 99 and 98%. Overall, the outcomes could introduce the spindle-shaped PbWO_4_ nanostructure (produced at power of 60 W for 10 min and with usage of maltose) as an efficacious substance for water contamination removal under visible light.

## 4 Conclusions

Shortly, a facile and swift sonochemical route was employed for creation of spindle-shaped PbWO_4_ nanostructure (a highly efficient photocatalyst for removal of organic pollution in water) with the aid of an environmentally friendly capping agent (maltose) for the first time. To optimize efficiency, dimension and structure of lead tungstate, the diverse effective factors were altered such as time, dose of precursors, power of ultrasound waves and kind of capping agents. The attributes of PbWO_4_ samples were checked with the aid of diverse identification techniques. The synthesized lead tungstate samples in role of visible-light photocatalyst were applied to remove organic pollution in water. Kind of pollutants, dose and type of catalyst as notable factors were examined in contaminant removal capability. Very favorable catalytic yield and durability demonstrated by spindle-shaped PbWO_4_ nanostructure (made at power of 60 W for 10 min and with usage of maltose). Usage of ultrasonic irradiation could bring to improvement of catalytic yield of PbWO_4_ to 93% in elimination of Acid Black 1. Overall, the outcomes could offer the spindle-shaped PbWO_4_ nanostructure as an efficacious substance to eliminate water contamination under visible light.

## CRediT authorship contribution statement

**Sahar Zinatloo-Ajabshir:** Formal analysis, Investigation, Data curation, Conceptualization, Methodology, Supervision, Project administration, Validation, Writing - review & editing, Writing - original draft, Resources. **Mahin Baladi:** Formal analysis, Investigation, Data curation, Validation, Methodology. **Masoud Salavati-Niasari:** Formal analysis, Investigation, Data curation, Writing - review & editing, Supervision, Project administration.

## Declaration of Competing Interest

The authors declare that they have no known competing financial interests or personal relationships that could have appeared to influence the work reported in this paper.
